# Supplementary material for: Cellobiohydrolase B of Aspergillus niger over-expressed in Pichia pastoris stimulates hydrolysis of oil palm empty fruit bunches
Source: PeerJ. 2017 Oct 12;5:e3909. doi: 10.7717/peerj.3909 (PMC5641429; doi:10.7717/peerj.3909)
Supplement: Data S1 — Raw data generated in this study. [file peerj-05-3909-s001.docx]

**Raw data presented and used in this work**

1. **cDNA sequence of *cbh*B**

The cDNA sequences of *cbh*B was deposited in the GenBank with an accession number of: KR052992.1

Webpage: <https://www.ncbi.nlm.nih.gov/nuccore/KR052992>

1. **Temperature and pH optima of CBHB (Fig.5)**

**Temp optima**

|  | Temp (oC) | 30 | 40 | 45 | 50 | 55 | 60 | 70 |
| --- | --- | --- | --- | --- | --- | --- | --- | --- |
| Velocity | Rep1 | 30600 | 33311 | 40131 | 41981 | 31521 | 12322 | 7921 |
| in FU | Rep2 | 31310 | 35302 | 41640 | 42983 | 30424 | 12764 | 7906 |
|  | Avg | 30955 | 34305 | 40885 | 42481 | 30970 | 12541 | 7912 |

Raw readings were then converted into relative activity with the highest reading assumed as 100%.

**pH optima**

|  |  |  |  | Phosphate buffer | | |
| --- | --- | --- | --- | --- | --- | --- |
|  | Citrate buffer | | | |  |  |
| **pH** | 3 | 4 | 5 | 6 | 7 | 8 |
| Rep1 | 2145 | 43155 | 16609 | 8074 |  |  |
| Rep2 | 2058 | 44469 | 17565 | 7824 |  |  |
| Avg | 2103 | 43812 | 17087 | 8324 |  |  |
| Rep1 |  |  |  | 9398 | 14082 | 1289 |
| Rep2 |  |  |  | 9880 | 14834 | 1339 |
| Avg |  |  |  | 9639 | 14458 | 1314 |

Raw readings were then converted into relative activity with the highest reading assumed as 100%.

1. **Temperature and pH stability of CBHB (Fig.5)**

**Temp stability**

| Residual activity (in FU) of CbhB after incubating at various temp for 30 mins | | | | | | | | | |
| --- | --- | --- | --- | --- | --- | --- | --- | --- | --- |
|  | Control | 30 | 40 | 45 | 50 | 55 | 60 | 70 | 80 |
| Rep1 | 46361 | 44905 | 44305 | 45486 | 44300 | 46076 | 44864 | 41369 | 42930 |
| Rep2 | 47122 | 46533 | 43937 | 43892 | 44346 | 43133 | 44190 | 41229 | 42752 |
| Avg | 46741.5 | 45719 | 44121 | 44689 | 44323 | 44604.5 | 44527 | 41299 | 42841 |

Raw readings were then converted into relative activity with the highest reading assumed as 100%.

**pH stability**

Residual activity (in FU) of CbhB after incubating at various pH for 30 mins

|  | Control | 3 | 4 | 5 | 6 | 7 | 8 | 9 | 10 |
| --- | --- | --- | --- | --- | --- | --- | --- | --- | --- |
| Rep1 | 35693 | 39589 | 41819 | 39673 | 40584 | 41942 | 41950 | 39631 | 37601 |
| Rep2 | 34987 | 41197 | 43963 | 41707 | 42153 | 44358 | 43389 | 41663 | 39043 |
| Avg | 35340 | 40393 | 42891 | 40690 | 41368.5 | 43150 | 42669.5 | 40647 | 38322 |

Raw readings were then converted into relative activity with the highest reading assumed as 100%.

1. **Kinetics assays for determination of K_m_ and V_max_ (Fig.6)**

|  |  | |  | |  | |  | |  |
| --- | --- | --- | --- | --- | --- | --- | --- | --- | --- |
|  | **MUC (mM)** | | 0.5 | | 1 | | 1.5 | | 2 |
| Velocity in FU | Rep1 | | 31113 | | 37719 | | 41901 | | 46161 |
|  | Rep2 | | 34154 | | 37005 | | 38486 | | 39974 |
|  | Rep3 | | 32640 | | 37401 | | 40171 | | 42960 |
|  | Avg | | 32636 | | 37375 | | 40186 | | 43032 |
|  | |  | |  | |  | |  | |
| 1/[S] (mM^-1^) | | 2 | | 1 | | 0.67 | | 0.5 | |
| 1/V (FU^-1^) | | 3.06E-05 | | 2.68E-05 | | 2.49E-05 | | 2.32E-05 | |

| Vmax: 50000 FU, or 31.8 nmol in a 200 uL microplate well.  So, in 400 uL rxn mix there was 63.6 nmol MU, or  Equal to 63.6nmol/15min/0.003mg,  = 1413 nmol/min/mg or  = 1.41 U/mg (Vmax)  Km: 0.25 mM |
| --- |

Conversion of FU into MU was based on the MU Standard Curve.

| MU (nmol) | 2 | 5 | 10 | 20 | 30 | 50 |
| --- | --- | --- | --- | --- | --- | --- |
| Rep1 | 1671 | 7415 | 13901 | 31180 | 48001 | 78510 |
| Rep2 | 1712 | 7620 | 13711 | 30930 | 47114 | 79510 |
| Avg | 1691.5 | 7517.5 | 13806 | 31055 | 47557.5 | 79010 |

1. **Effects of metal ions/reagent (Fig.7)**

|  | Ctrl | CaCl2 1mm | CaCl2 10mM | KCL 1mM | KCL 10mM | NaCl 1mM | NaCL 10 mM | ZnSO4 1mM | ZnSO4 10mM |
| --- | --- | --- | --- | --- | --- | --- | --- | --- | --- |
|  | 43707 | 43476 | 41900 | 42581 | 42694 | 42693 | 43740 | 45236 | 39209 |
|  | 43299 | 42251 | 40880 | 42158 | 43556 | 41196 | 41828 | 42890 | 39244 |
| Avg | 43503 | 42863.5 | 41390 | 42369.5 | 43125 | 41944.5 | 42784 | 44063 | 39226.5 |
|  |  |  |  |  |  |  |  |  |  |
|  | Ctrl no ion | CoCl2 1mM | CoCl2 10mM | BaCl2 1mM | BaCl2 10mM | CuSO4 1mM | CuSO4 10mM | MgSO4 1mM | MgSO4 10mM |
|  | 44617 | 40166 | 38585 | 43855 | 34825 | 42659 | 32162 | 41086 | 40694 |
|  | 43151 | 40801 | 39585 | 43655 | 35020 | 42702 | 34030 | 40862 | 39834 |
| Avg | 43884 | 40483.5 | 39085 | 43755 | 34922.5 | 42680.5 | 33096 | 40974 | 40264 |
|  |  |  |  |  |  |  |  |  |  |
|  | MnCl2 1mM | MnCl2 10mM | Fe2SO4 1mM | Fe2SO4 10mM | EDTA 1mM | EDTA 10mM | Urea 0.1M | Urea 1M |  |
|  | 40154 | 36785 | 23467 | 3957 | 40942 | 42758 | 42537 | 41038 |  |
|  | 40514 | 36582 | 23532 | 3816 | 40942 | 43118 | 41770 | 40715 |  |
| Avg | 40334 | 36683.5 | 23499.5 | 3886.5 | 40942 | 42938 | 42153.5 | 40876.5 |  |
|  |  |  |  |  |  |  |  |  |  |

1. **ATR FT-IR profiles of OPEFB samples (Fig.9)**

As attached as FTIR.rawdata2 in the supplemental file upload.

1. **RSM models**

The raw data generated in RSM optimisation (13 sets) was presented in Table 3
