# Supplementary figures and images for: Cellobiohydrolase B of Aspergillus niger over-expressed in Pichia pastoris stimulates hydrolysis of oil palm empty fruit bunches
Source: PeerJ. 2017 Oct 12;5:e3909. doi: 10.7717/peerj.3909 (PMC5641429; doi:10.7717/peerj.3909)

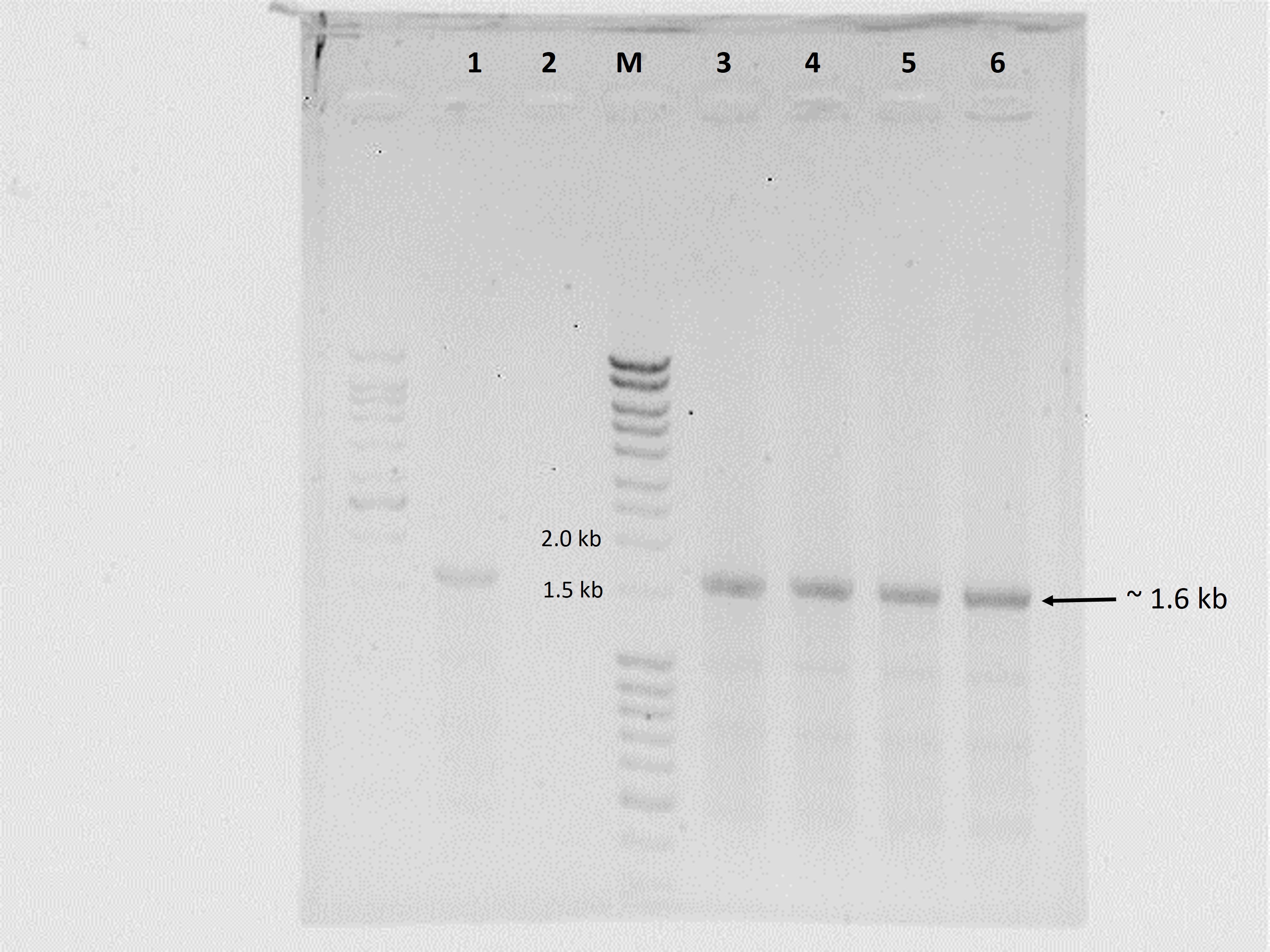

Supplement: Figure S1 [file peerj-05-3909-s003.jpg]

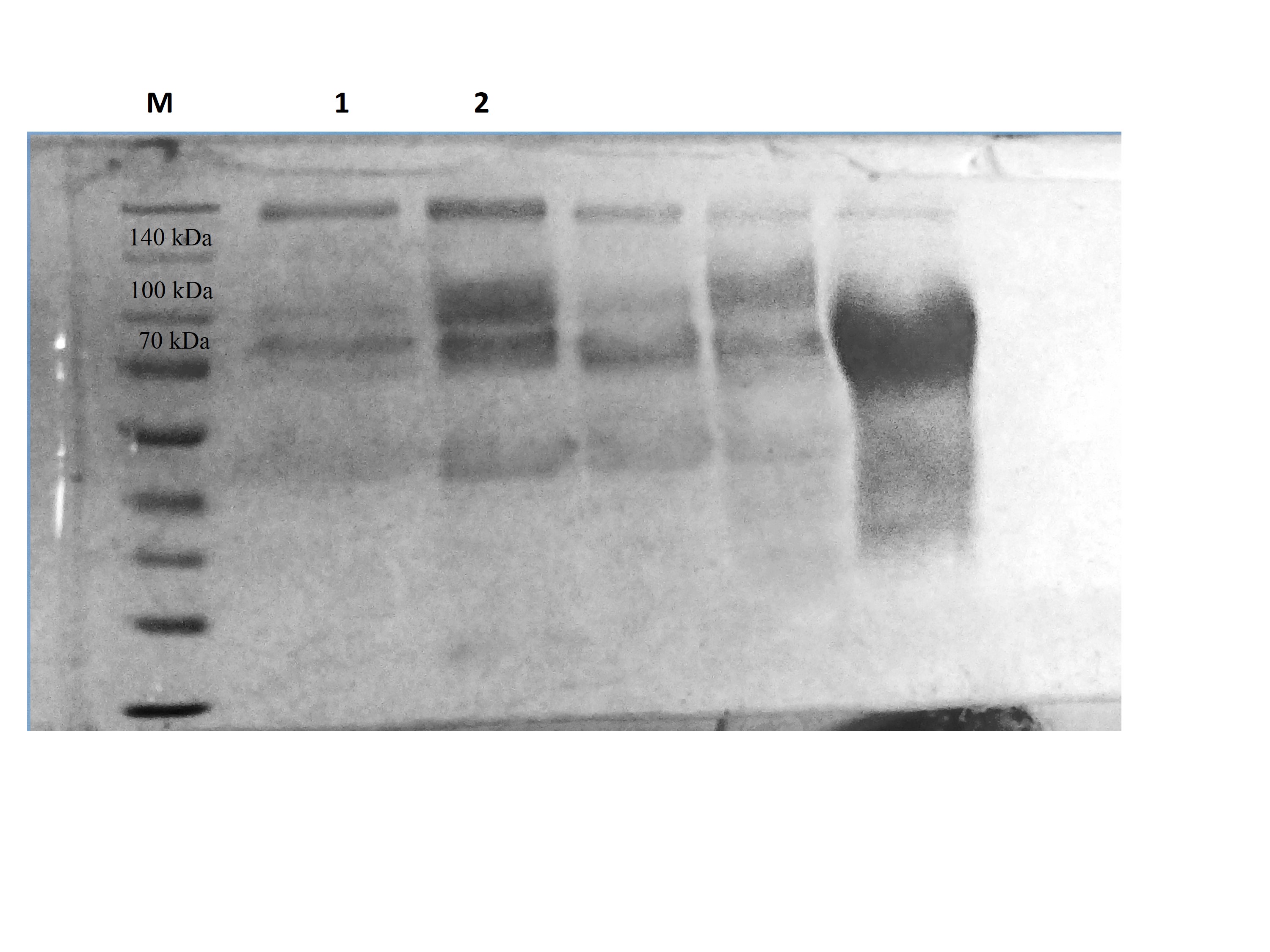

Supplement: Figure S2 [file peerj-05-3909-s004.jpg]

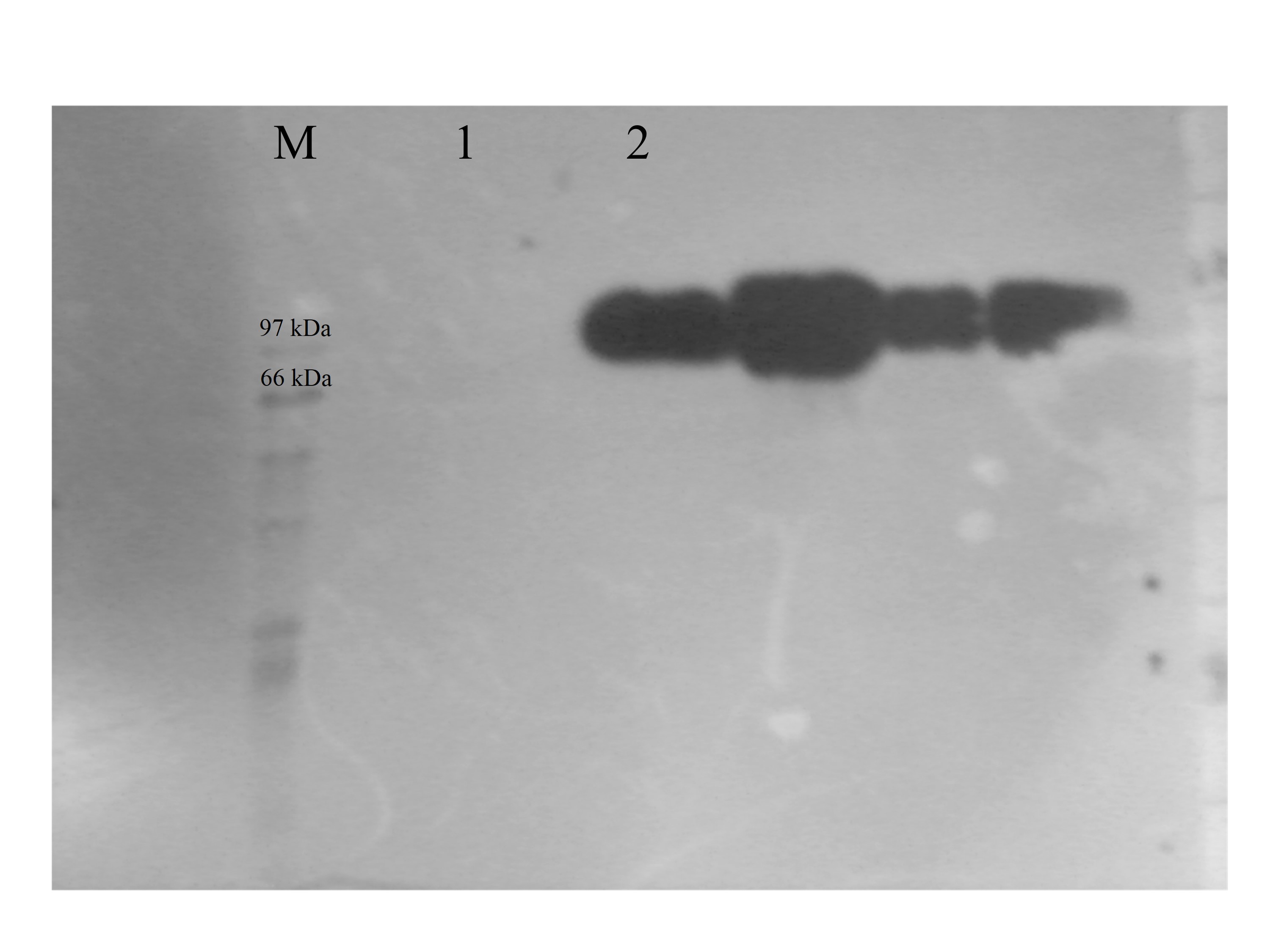

Supplement: Figure S3 [file peerj-05-3909-s005.jpg]

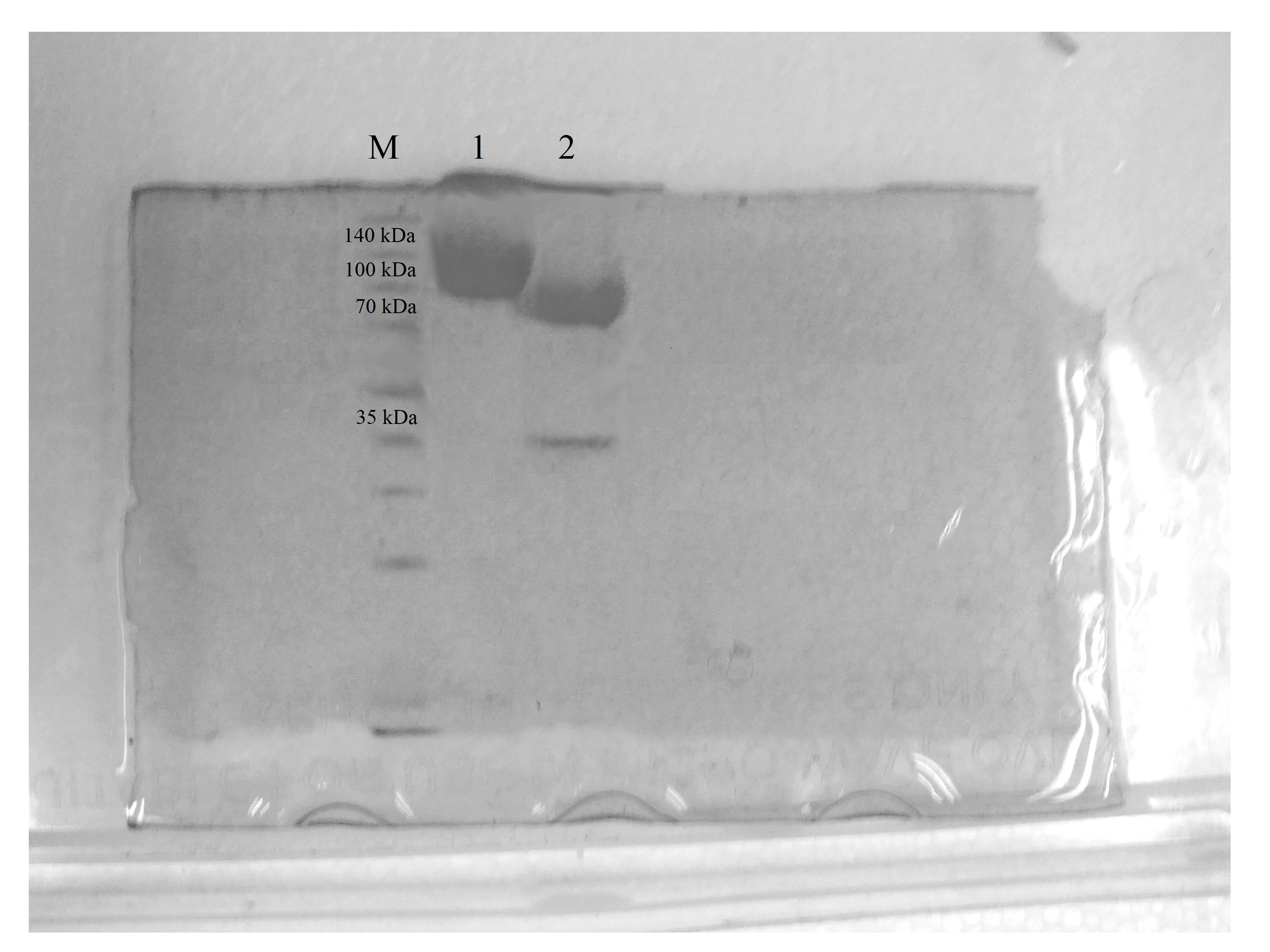

Supplement: Figure S4 [file peerj-05-3909-s006.jpg]
